# Muscle Synergies Heavily Influence the Neural Control of Arm Endpoint Stiffness and Energy Consumption

**DOI:** 10.1371/journal.pcbi.1004737

**Published:** 2016-02-11

**Authors:** Joshua M. Inouye, Francisco J. Valero-Cuevas

**Affiliations:** 1 Department of Biomedical Engineering, University of Virginia, Charlottesville, Virginia, United States of America; 2 Department of Biomedical Engineering, University of Southern California, Los Angeles, California, United States of America; 3 Department of Biokinesiology and Physical Therapy, University of Southern California, Los Angeles, California, United States of America; University College London, UNITED KINGDOM

## Abstract

Much debate has arisen from research on muscle synergies with respect to both limb impedance control and energy consumption. Studies of limb impedance control in the context of reaching movements and postural tasks have produced divergent findings, and this study explores whether the use of synergies by the central nervous system (CNS) can resolve these findings and also provide insights on mechanisms of energy consumption. In this study, we phrase these debates at the conceptual level of interactions between neural degrees of freedom and tasks constraints. This allows us to examine the ability of experimentally-observed synergies—correlated muscle activations—to control both energy consumption and the stiffness component of limb endpoint impedance. In our nominal 6-muscle planar arm model, muscle synergies and the desired size, shape, and orientation of endpoint stiffness ellipses, are expressed as linear constraints that define the set of feasible muscle activation patterns. Quadratic programming allows us to predict whether and how energy consumption can be minimized throughout the workspace of the limb given those linear constraints. We show that the presence of synergies drastically decreases the ability of the CNS to vary the properties of the endpoint stiffness and can even preclude the ability to minimize energy. Furthermore, the capacity to minimize energy consumption—when available—can be greatly affected by arm posture. Our computational approach helps reconcile divergent findings and conclusions about task-specific regulation of endpoint stiffness and energy consumption in the context of synergies. But more generally, these results provide further evidence that the benefits and disadvantages of muscle synergies go hand-in-hand with the structure of feasible muscle activation patterns afforded by the mechanics of the limb and task constraints. These insights will help design experiments to elucidate the interplay between synergies and the mechanisms of learning, plasticity, versatility and pathology in neuromuscular systems.

## Author Summary

The manner in which the nervous system coordinates the multiple muscles in the body is complex. It has been studied for decades, but a more full understanding is needed to enable the development of effective evaluation and treatment methods in disorders that cause neuromuscular disability such as cerebral palsy and stroke. In addition, the computational control of robots has and will continue to improve as the brain’s methods of muscular control are progressively reverse-engineered. Here, we study the capacity of arm muscles to regulate the stiffness of the hand for tasks such as using tools, stabilizing hand-held objects, and using doors. Using a simplified but generalizable model, we show that there will be necessary trade-offs in the functional capabilities of the limb if the nervous system chooses to control muscles in functional groups. This adds to our understanding of the consequences of different strategies to control muscles for real-world tasks with multiple and often competing demands. It enables future research and clinical experiments on the learning and execution of the multiple tasks of varying difficulty encountered in real life. It also sheds light on the design of control strategies for robots to operate in human and unstructured environments.

## Introduction

Limb impedance control by the central nervous system (CNS) has been a subject of much study and debate over the past three decades. Numerous experiments and theoretical analyses have studied the biomechanical and neuromuscular capabilities of the CNS to regulate the impedance of a limb (e.g., [1–22]). The preferred paradigm of many studies is to analyze the stiffness the human arm can produce at its endpoint (i.e., the hand) in reaching-like postures in a horizontal plane in front of a seated subject. One set of experimental findings is that, after some training, the CNS can regulate to varying degrees the orientation and eccentricity of arm stiffness ellipses to perform a task more reliably and efficiently than before training [1, 4, 5, 10]. Another set of experiments concludes that the CNS cannot arbitrarily regulate endpoint stiffness, and that it is only able to rotate the orientation of the stiffness ellipsoid around 30° [15, 20, 21]. Here we focus on reconciling some of these conflicting results by using novel computational analyses of tendon-driven systems to establish the neuromechanical capabilities of biological limbs in the context of muscle synergies.

The existence and interpretation of muscle synergies is controversial and has received much attention in the recent literature [23–29]. Synergies—defined as the correlated activation of multiple muscles by using a small number of coordination patterns—are theoretically one way to simplify the control of movement in the highly redundant musculature of vertebrates. They have also been observed by EMG measurements during reaching movements with the arm [19]. Here we explore the restrictions synergies could impose on the ability of the CNS to synthesize arm endpoint stiffnesses with differing characteristics.

There is extensive literature on the analysis and synthesis of endpoint stiffness in robotic limbs [7, 30–33]. The theoretical contributions and conclusions of these robotics studies are independent of the mechanisms and limitations of sensorimotor control by the CNS, and hence form a good theoretical foundation to design and interpret experiments to study the neuromechanical capabilities of biological limbs both in the presence and absence of synergies. In [34] such an approach was used to compare theoretical predictions against experimental findings by recording from a few finger muscles.

**Fig 1:**
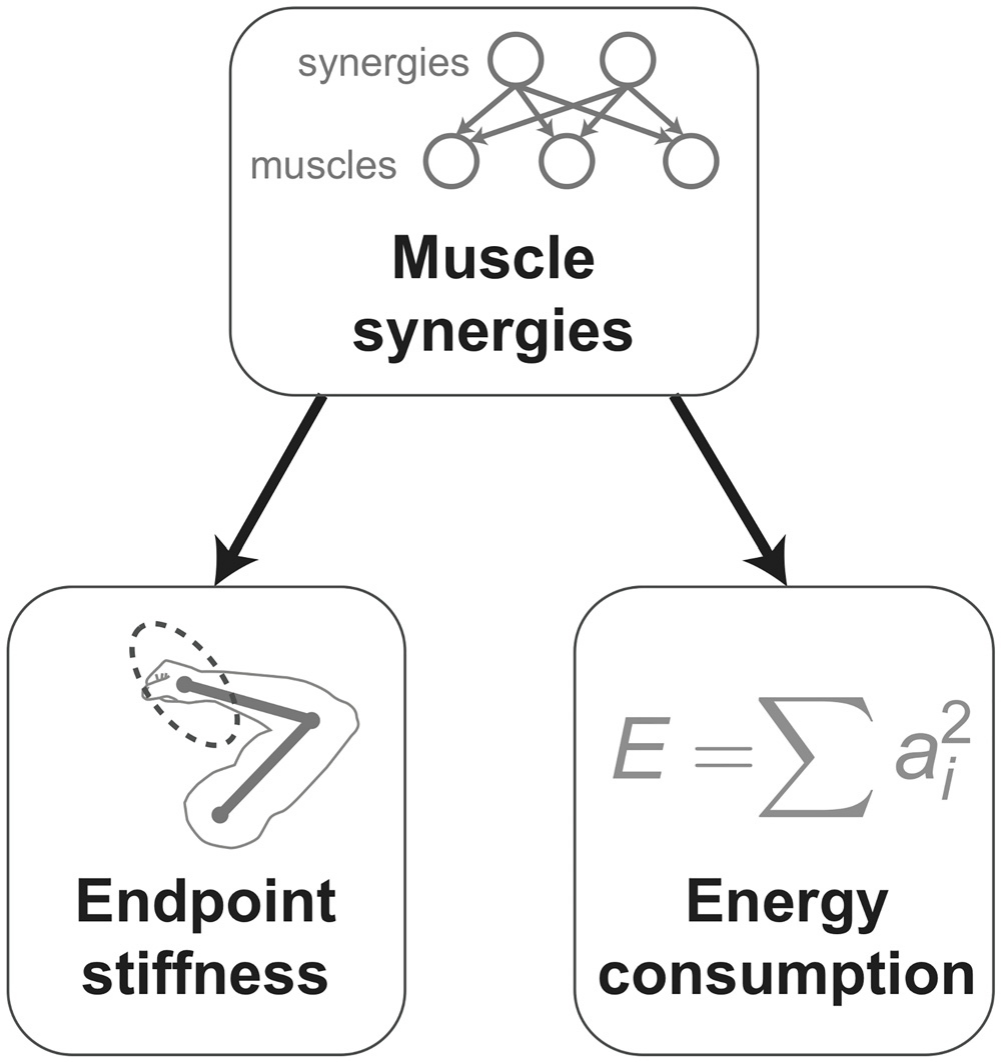
Overview. We explore the interactions of muscle synergies with endpoint stiffness synthesis and energy consumption in the context of feasible activations to meet task constraints.

In this study, we investigate the effects of muscle synergies on endpoint stiffness synthesis and energy consumption (Fig 1). To this end, we apply principles of robotics in a novel computational formulation for tendon-driven systems that allows us to easily and efficiently analyze the range over which the stiffness of the endpoint of the limb can be modified. More specifically, we are referring to the magnitude of endpoint stiffness in a variety of directions which can be mathematically approximated by a stiffness ellipse. From an engineering perspective, we can call this the range of ‘stiffness realizations’ because each of them is an instance of the neuromechanical capabilities of the limb. By studying stiffness realizations in the presence and absence of muscle synergies throughout the workspace, we find that synergies drastically decrease the ability of the CNS to synthesize an arbitrary stiffness ellipse.

Importantly, our work takes on the approach that we are interested in finding the families of feasible endpoint stiffness realizations throughout the workspace of the limb. That is, how much can the nervous system control the size, shape (i.e., eccentricity) and orientation of endpoint stiffness ellipses for all limb postures? Due to muscle redundancy, there may be multiple ways to achieve any one possible stiffness ellipse. That set of multiple neural commands that can achieve a given realization is its ‘*feasible activation set*’ [35, 36]. We can therefore optimize over that set to find the muscle activation pattern that produces the desired endpoint stiffness while minimizing energy consumption. The question is, then, how do muscle synergies compromise—or even annihilate—the ability of the nervous system to control the properties of endpoint stiffness and minimize energy consumption?

As mentioned in the Discussion, this neuromechanical approach emphasizes the feasibility of neuromechanical actions and allows us to consider several potential confounds when comparing across studies. Such studies may include examination of the extent, efficacy and nature of training, the influence of limb postures on task goals specified by the experimenter, and the implicit neural strategies specified by the CNS with regard to stiffness regulation in health and disease. Our results also allow us to discuss how learning, experimental design, and neural strategies affect our ability to tune endpoint stiffness.

## Methods

### Arm model

**Fig 2:**
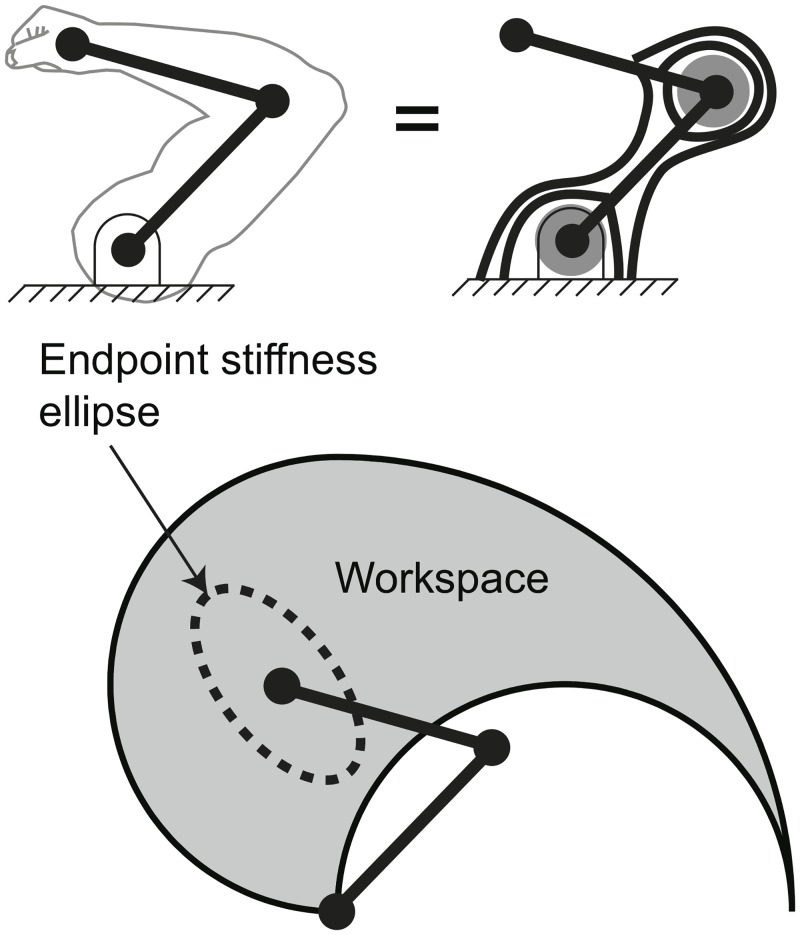
Methods. We use a 6-muscle planar arm model to quantify the effects of synergies on endpoint stiffness and energy consumption within the workspace. For any posture in the workspace of the arm, neural commands to the muscles can set the active endpoint stiffness of the limb—visualized as an ellipse of a particular size, shape (i.e., eccentricity), and orientation.

We use a simplified planar arm model with 6 muscles similar to those that have been used in other theoretical and computational studies [3, 8, 12, 13, 37] (Fig 2). In those studies as well as this study—as described below—the spatial distribution of the stiffness of the endpoint of the limb, *K*_*end*_, is a matrix that is calculated as a function of individual musculotendon stiffnesses, which are the elements of the matrix *K*_*muscles*_. The individual musculotendon stiffness generated by each muscle is represented by a numerical variable that is, in effect, a lumped parameter model introduced by Hogan and Mussa-Ivaldi [8, 13] that combines the active and passive components of muscle and the passive components of tendon. This approximation remains commonplace and valid in the computational literature whose goal is not to simulate the physiology of musculotendon stiffness, but rather use a mechanical analogue of musculotendons to allow the study of the feasible mechanical behavior of the limb. This lumped parameter approach is accepted in the computational literature to replicate the fact that musculotendons have stiffness, and that stiffness can be modulated by the individual neural commands to the muscles of a limb, the activation vector $\vec{a}$. The reader is referred to the literature for details [3, 8, 12, 13, 37], but a brief description is presented below.

We use workspace constraints identical to those used in [8] to produce the workspace of the limb (i.e., the locations that are reachable by the endpoint), also shown in Fig 2. As is common, we use singular value decomposition (SVD) to transform the endpoint stiffness matrix (*K*_*end*_) to an ellipse that represents the characteristics of this matrix—shape (can also be termed eccentricity)—measured by the matrix condition number, and orientation of the major axis with respect to the x-axis.

### Theoretical endpoint stiffness formulation

We begin our formulation with the endpoint stiffness matrix, *K*_*end*_, as a function of muscle active stiffnesses, *K*_*muscles*_. As a point of clarification, we focus on muscle active stiffness that results from the feedforward activation of the muscle such as during force production or co-contraction. We do not include passive stiffness, which normally refers to the inherent material properties of the limb or muscles with no muscle activation. This could arise, for example, from tendon properties. Similarly, as mentioned in the Discussion, we do not include the time-delayed stiffness resulting from reflexes, often called reflexive stiffness. In our formulation, *K*_*end*_ relates the vector of differential endpoint displacements to differential endpoint forces:

$$\partial \vec{F}=K_{end}\partial \vec{x}$$ (1)

where $\partial \vec{F}$ is the endpoint force vector resulting from a displacement vector $\partial \vec{x}$. The joint stiffness matrix, *K*_*joint*_, relates the vector of differential joint angle displacements to differential joint torques:

$$\partial \vec{\tau }=K_{joint}\partial \vec{\theta }$$ (2)

where $\partial \vec{\tau }$ is the joint torque vector resulting from a joint angle displacement vector $\partial \vec{\theta }$. The endpoint stiffness matrix is dependent on the joint stiffness matrix as well as the manipulator Jacobian *J* (which is posture dependent: a vector of joint angles $\vec{\theta }$ uniquely defines the posture):

$$\dot{\vec{x}}=J\left(\vec{\theta }\right)\dot{\vec{\theta }}$$ (3)

where $\dot{\vec{x}}$ denotes the endpoint velocity vector and $\dot{\vec{\theta }}$ denotes the joint angle velocity vector.

The endpoint stiffness matrix, in the absence of an external tip force, is given by [8]:

$$K_{end}=J^{-T}K_{joint}J^{-1}$$ (4)

Furthermore, the joint stiffness matrix is given by [8]:

$$K_{joint}=RK_{muscles}R^{T}$$ (5)

where *K*_*muscles*_ is the diagonal matrix of muscle stiffnesses and *R* is the moment arm matrix relating joint angle changes to tendon displacements, $\partial \vec{s}$:

$$\partial \vec{s}=R\partial \vec{\theta }$$ (6)

Combining Eqs 4 and 5, we obtain the relationship of muscle stiffness to endpoint stiffness:

$$K_{end}=J^{-T}RK_{muscles}R^{T}J^{-1}$$ (7)

This is equivalent to other formulations, such as in [34]. The diagonal elements of *K*_*muscles*_ are assumed to be linearly related to their corresponding muscle forces [38]:

$$K_{muscles}=\alpha \times diag\left({\vec{F}}_{muscles}\right)$$ (8)

For simplicity in this study, we assume the scaling factor *α* is equal to one. We can define a diagonal matrix of maximal muscle forces, *F*_*max*_, so that we can calculate ${\vec{F}}_{muscles}$ using the muscle activation vector $\vec{a}$:

$${\vec{F}}_{muscles}=F_{max}\vec{a}$$ (9)

The entries of $\vec{a}$ are inside the interval [0, 1] since muscle force can only be positive (this constraint can also be expressed as the requirement that the activation vector lies in the positive orthant of the unit hypercube in activation space). We assume *F*_*max*_ to be the identity matrix for simplicity in this study.

### Reformulation of endpoint stiffness as a set of linear equations

Using Eqs 7, 8 and 9 and reformulating the endpoint stiffness matrix, the moment arm matrix, and the Jacobian, we can make the endpoint stiffness ${\tilde{K}}_{end}$ a vector that is a linear function of the muscle activations.

$${\tilde{K}}_{end}={\tilde{J}}^{-T}\tilde{R}F_{max}\vec{a}$$ (10)

**Fig 3:**
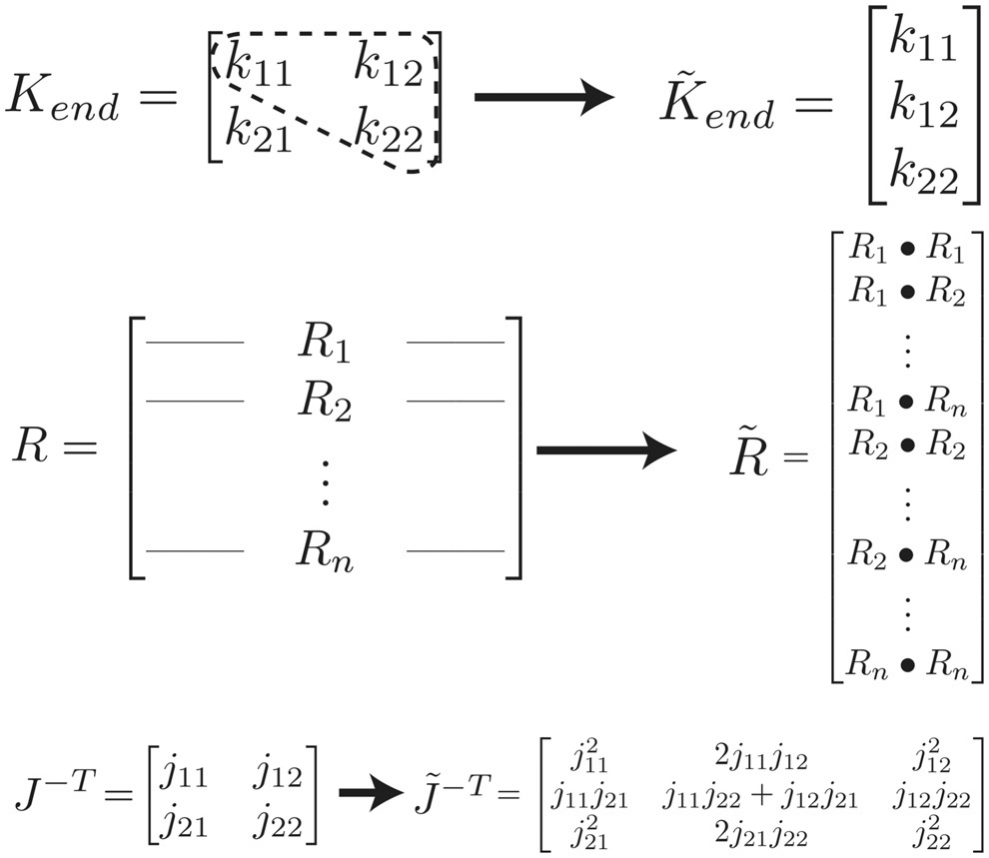
Reformulation of stiffness equations. Obtaining Eq 10 requires that we transform the matrices in Eq 7. In this way, the vector ${\tilde{K}}_{end}$ can be expressed as a set of linear equations in $\vec{a}$. These linear equations become the linear constraints for endpoint stiffness that we use to solve the quadratic programming problem to minimize energy. (•) denotes element-by-element multiplication, and *R*_*i*_ is the *i*^*th*^ row of *R*.

We show these reformulations in Fig 3. (•) denotes element-by-element multiplication, and *R*_*i*_ is the *i*^*th*^ row of *R*. The Jacobian reformulation is specific to the 2-link planar arm model, but similar expressions can be formulated for Jacobians of higher dimensions.

The endpoint stiffness and the moment arm matrices have been previously reformulated in this way [33]. And [34] speaks of the equations defining iso-stiffness planes. But to the best of our knowledge, no study has yet reformulated the Jacobian in this way to allow for the simple set of linear equations found in Eq 10 relating muscle activations to endpoint stiffness.

### Energy consumption

Each realization of a given endpoint stiffness matrix—and its associated ellipse—is produced by a given neural command, $\vec{a}$, as shown in Eq 10. As per Eq 9, the individual forces in each muscle contribute to the overall stiffness of the limb while producing zero net torque at each joint to maintain equilibrium. These isometric muscle forces have a metabolic cost, which we calculate as the sum of squares of muscle forces [39]:

$$energy=\sum_{k=1}^{6}{\left(F_{max_{k}}*a_{k}\right)}^{2}$$ (11)

### Simulating synergies

**Fig 4:**
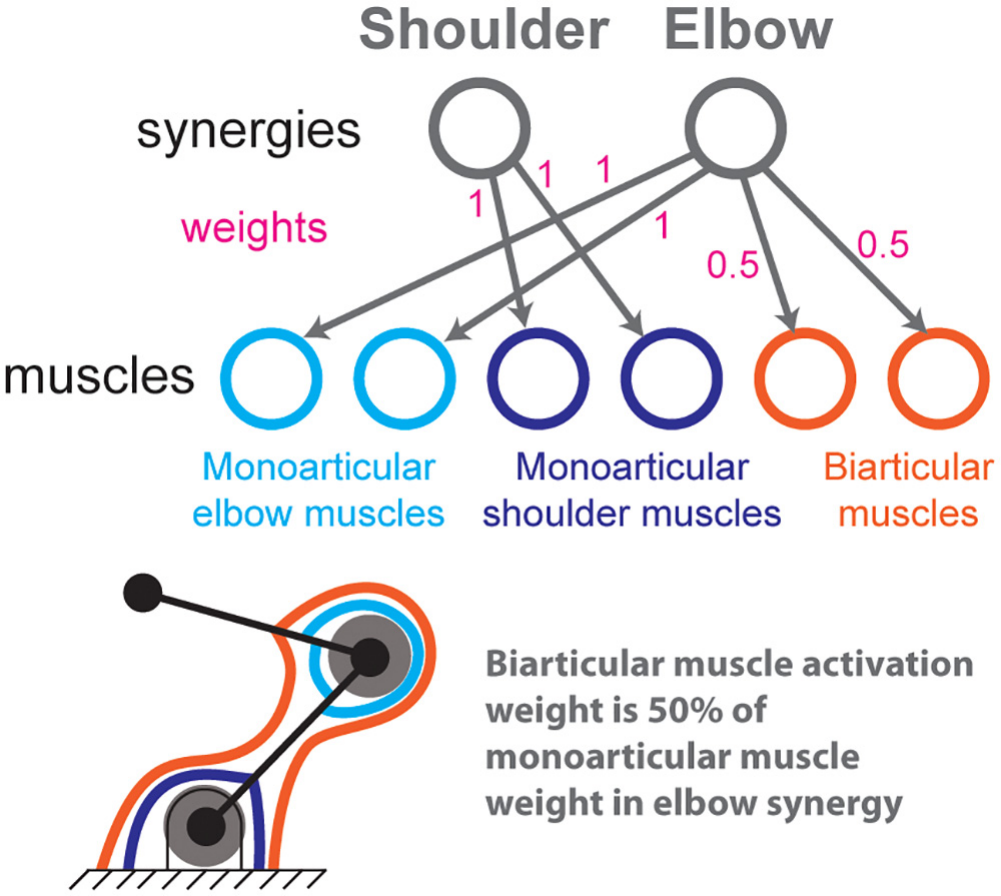
Shoulder and elbow synergies. We simulate experimentally-observed synergies that group the mono-articular shoulder muscles and all the muscles crossing the elbow. Briefly, a synergy is the correlated activity of muscle activations. Each synergy is independently controlled and synergistically drives its muscles according to specific muscle weighting parameters. In this case, we simulate how two synergies drive the six muscles of an arm as per the number of synergies and weights reported by [19].

We simulate synergies that have been experimentally observed in a previous EMG study of static postures similar to those used during arm reaching tasks [19]. These synergies couple the bi-articular muscles with the mono-articular elbow muscles as shown in Fig 4. Quantitatively, that study found that the elbow stiffness from co-contraction of the bi-articular muscles was approximately one half of the elbow stiffness from the mono-articular elbow muscles. They did not find mono-articular shoulder muscles to have synergies with the bi-articular muscles.

Consequently, for our model, the activation of the shoulder synergy, *a*_*shoulder*_, activated the two mono-articular shoulder muscles with unity weight. The activation of the the elbow synergy, *a*_*elbow*_, activated the mono-articular elbow muscles with unity weight and the biarticular muscles with weights of one-half (Fig 4).

In the presence of these synergies, one parameter suffices to change the orientation and shape of the endpoint stiffness ellipse: the ratio of elbow synergy activation to shoulder synergy activation, *a*_*elbow*_/*a*_*shoulder*_. Increasing the activation of both synergies simultaneously and proportionately only increases the size of the ellipse but not its shape or orientation (i.e., the angle from the x-axis to the major axis of the ellipse). As we will see in the results, this one-dimensional manifold in muscle activation space does not allow the realization of the arbitrary endpoint stiffness ellipses because the synergies, by coupling muscles, also couple two important stiffness characteristics: the stiffness ellipse’s shape and orientation. That is, in the presence of synergies, as just described, there is only one free parameter that can be varied to control these characteristics, *a*_*elbow*_/*a*_*shoulder*_. Therefore, changing orientation independently of shape is impossible.

To further explore this coupling of task constraints by synergies, we vary the ratio of shoulder synergy activation to elbow synergy activation (by varying *a*_*elbow*_/*a*_*shoulder*_) over a range of 2 orders of magnitude (1/10 to 10) to see how much the orientation of the ellipse is able to change.

### Checking for realizable endpoint stiffnesses and considering energy minimization

In the absence of synergies in our model (i..e, all muscles can be activated independently), we can determine if the arm is able to meet the constraints that

1. all activation vectors lie within a unit 6-dimensional cube in the positive octant of the activation space [35, 40] (i..e, all activations lie between 0 and 1)
  $$0\leq a_{i}\leq 1$$ (12)
2. the net joint torque vector is zero (i.e., the posture is in equilibrium)
  $$RF_{max}\vec{a}=0$$ (13)
3. and the endpoint stiffness has a given desired shape and orientation, as in Eq 10
  $${\tilde{J}}^{-T}\tilde{R}F_{max}\vec{a}={\tilde{K}}_{end,desired}$$ (14)

Then if $\exists \;\vec{a}$ s.t. Eqs 14, 13 and 12, are satisfied, ${\tilde{K}}_{end,desired}$ is realizable in the absence of synergies.

**Fig 5:**
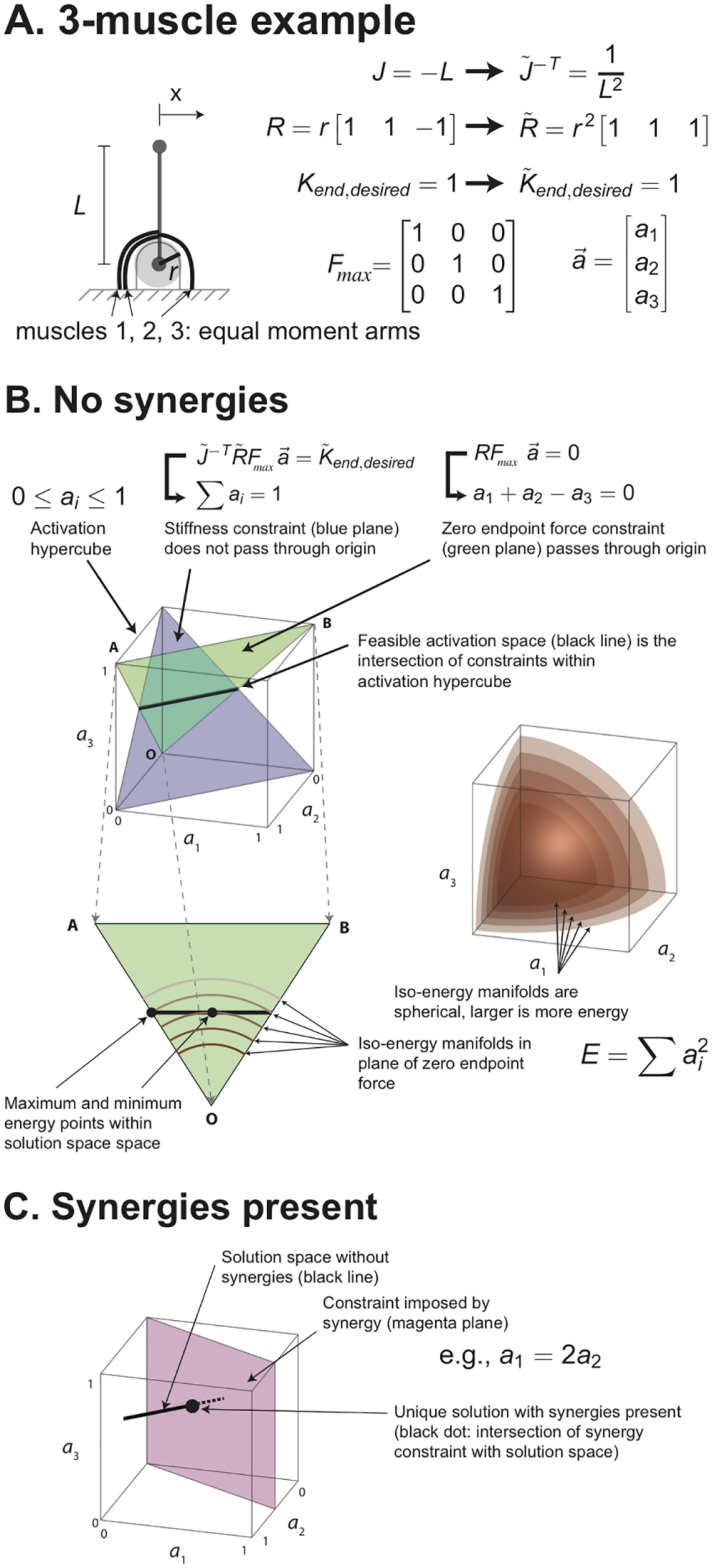
Visualization of mechanical and energetic constraints. **A**. Consider a limb with one rotational joint driven by 3 muscles. We show the parameters and variables needed to implement Eq 14. **B**. As described elsewhere in detail [34–36, 41, 42], the set of all physiologically feasible neural commands to a limb that can be visualized as a positive hypercube of as many dimensions as there are independently controllable muscles (3 in this case). Each constraint that defines the mechanical task reduces the set of feasible neural commands to a subset of that hypercube. Therefore, the feasible activation set for a task is the intersection of those constraints that lie within the hypercube. If the constraints are linear functions of activation, they reduce the subset of feasible neural commands to a hyperplane. We see this for the linear constraints defining the size, shape (i.e., eccentricity) and orientation of the desired endpoint stiffness ellipse (blue plane); and enforcing static equilibrium with zero endpoint force (green plane). Their intersections with the hypercube are shown schematically as triangular polygons. The feasible activation set that meets both constraints, if it exists, is the black line—which naturally contains infinitely many points (i.e., muscle redundancy). The individual solution with the minimal energetic cost is given by the point where the line is tangential to the smallest spherical manifold defined by the quadratic cost function in Eq 11. **C**. A muscle synergy can similarly be visualized as a constraint that ties the activations of several muscles in an obligatory way, which in this 3-dimensional example reduces the set of all physiologically feasible neural commands from a cube to a plane. This reduction in independently controllable muscle actions has the inevitable consequence of reducing—or even annihilating—the ability to meet multiple endpoint stiffness and energetic constrains as shown in our results.

An illustration of these constraints, the existence of a solution, and the potential for energy minimization in the absence of synergies is illustrated in Fig 5 for a simple 3-muscle model. We use three muscles because this allows us to visualize the feasible activation space in 3D, and each of the linear constraints can be shown as a plane, whose intersection is a line that still holds some redundancy. Since this example is for a manipulator with only one joint, the endpoint stiffness is only in the x-direction. Thus

1. the feasible activation set begins as a 3-dimensional unit cube in the positive octant.
2. The constraint of zero endpoint force is a 2-dimensional plane in activation space passing through the origin. This is because endpoint forces have a minimal value of 0 at zero activation).
3. The constraint for desired endpoint stiffness of unity is also a 2-dimensional plane in activation space, but it does not pass through the origin. This is because muscle activation is required to produce stiffness: at the origin, there is no muscle contraction, therefore there is no muscle stiffness stiffness or endpoint stiffness.

This geometric interpretation [34, 35, 43, 44] helps us understand the effect of synergies as additional constraints on feasible activations. The intersection of the first two functional constraints is a one-dimensional linear subspace of solutions that mathematically satisfy Eqs 14 and 13. Further constraining this subspace by the activation N-cube (Eq 12) results in the muscle activation solution space to realize a unity stiffness. In this the feasible activation space, that has the structure of a one-dimensional subspace (i.e., a line), energy (measured by the sum of the squares of the muscle forces, or concentric spheres) can be minimized or maximized by varying the activation point in the feasible activation set (i.e., a point along the line). The presence of even a simple synergy for this model (*a*_1_ = 2*a*_2_) results in an additional constraint plane that passes though the origin that will reduce the feasible activation set, reducing the dimensionality of the solution space. In this simple example, the dimensionality is reduced to zero—a unique solution [40, 43, 45]. But even in high dimensions [41], synergies will reduce what is already a well-structured space.

In our 6-muscle model, the activation hypercube is 6-dimensional. The constraint of zero endpoint force is a 4-dimensional hyperplane in activation space passing through the origin (6 dimensions − 2 equilibrium constraints = 4-dimensional solution space; Eq 12 is a system of two equations, one for each joint). The constraint for a desired endpoint stiffness is a 3-dimensional hyperplane in activation space (6 dimensions − 3 stiffness constraints = 3-dimensional solution space; as per Fig 3, Eq 14 is a system of three equations, one for each unique element of the symmetric matrix ${\tilde{K}}_{end,desired}$). The intersection of these two hyperplanes is a one-dimensional linear subspace (6 dimensions − 2 equilibrium constraints − 3 stiffness constraints = 1-dimensional feasible activation space) embedded in 6-dimensional space. It satisfies Eqs 14 and 13. If any part of this solution subspace lies in the activation N-cube (satisfying Eq 12 as well), then the desired stiffness is realizable. Furthermore, synergy constraints can reduce the dimensionality of the solution space to zero (i.e., there is a solution, it will be the unique solution of a point at the intersection of a line with a plane), or they can overconstrain the problem, making the desired stiffness unrealizable.

**Fig 6:**
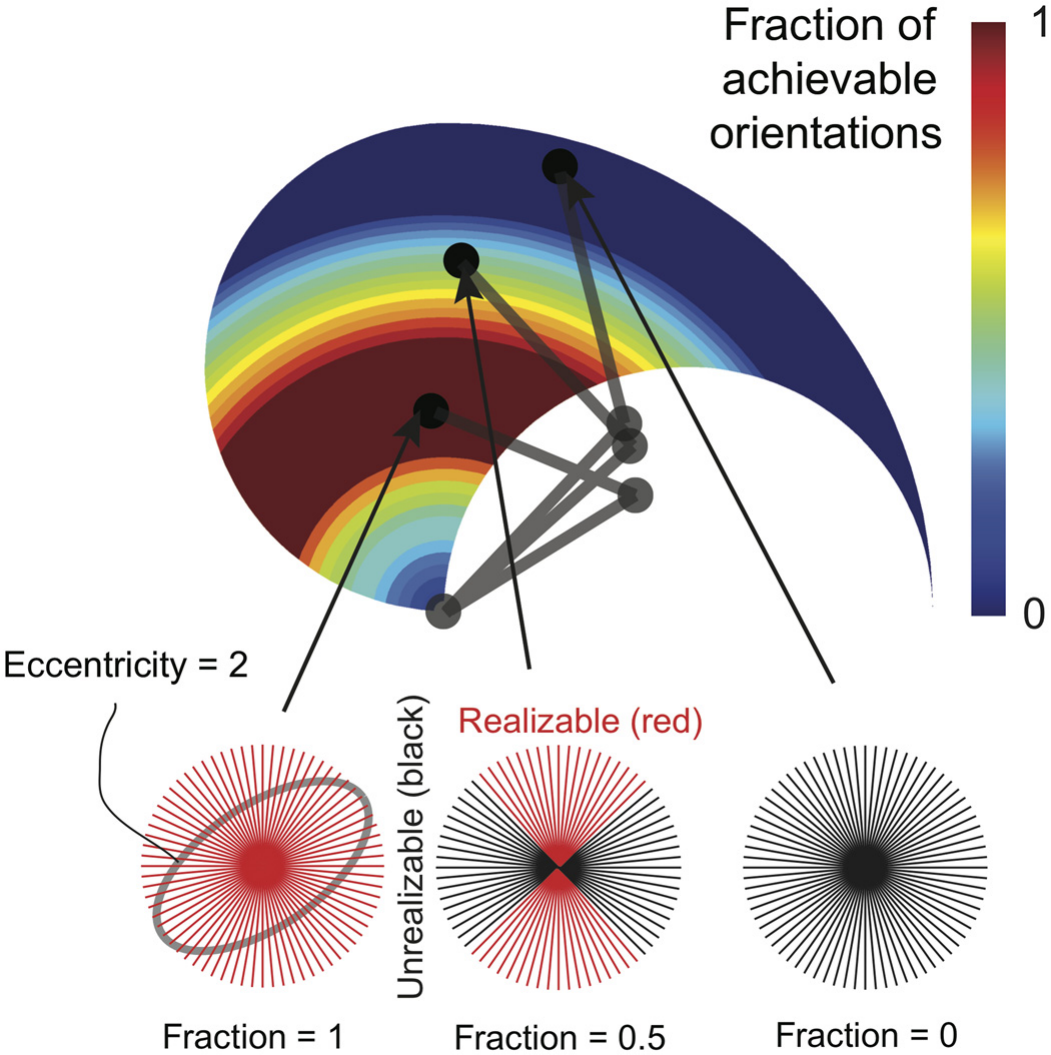
Method to find realizable endpoint stiffnesses throughout the workspace. Using our 6-muscle planar arm model, we are able to iteratively check for realizable endpoint stiffness ellipses throughout the workspace. In this case, the ability to set its orientation in 5 degree increments for a given stiffness ellipse shape (i.e., eccentricity). As the posture of the limb changes to make the endpoint visit each point in the workspace, the associated changes in its Jacobian matrix and the constraints of the task interact to affect the realizable endpoint stiffness ellipses. Red regions indicate the locations of the endpoint where all orientations are realizable (i.e., 100% or a fraction of 1), whereas deep blue and black regions indicate the locations in the workspace for which there is very limited or non-existent ability to arbitrarily control the orientation of the endpoint stiffness ellipse (i.e., 20%—0% or fractions of 0.2—0).

Within this context, we can now explore the range of achievable endpoint stiffness ellipse orientations given the arm posture and a desired ellipse shape. To this end, we fixed both the condition number of the stiffness matrix and the posture, and then determined a set of desired endpoint stiffnesses, each corresponding to a different ellipse orientation. We formulated a constrained quadratic programming problem, with the optimization criteria being minimizing the sum of squares of muscle activations. If an optimum was found, then the orientation (for that specific posture and ellipse shape) is realizable. We did this every 5° around the full range of orientations (i.e., 180°) and then checked the fraction of these orientations that are realizable. An example of the fraction of realizable orientations for all postures in the workspace is shown in Fig 6.

### Exploring energy expenditure within the solution space

The constraints in the realizability tests have five equality constraints (Eqs 14 and 13). Since there are 6 muscles, if there is any solution which satisfies Eq 12, in general there will be a one-dimensional feasible activation space for the desired endpoint stiffness embedded the 6-dimensional muscle activation space. Vertex enumeration algorithms can be used to determine the vertices of this one-dimensional manifold (which is a convex set [35]). However, we and the available literature, are also interested in the maximal and minimal energy expenditures within this feasible activation space. Therefore, we can use opposite quadratic programming optimization criteria to determine both of these energy expenditures. For the minimal energy expenditure, as already described, our optimization criteria is to minimize the sum of squares of the muscle forces. For maximal energy expenditure, our optimization criteria is to maximize the sum of squares of the muscle forces. From these extreme values we can then determine the maximal amount of energy reduction that is possible. For example, if the maximal energy expenditure is 0.5, say, and the minimal energy expenditure is 0.35, then there is a maximum of 30% reduction in energy possible.

Our rationale for quantifying these ratios is that, for given observed stiffness ellipsoid in human subjects experiments, we want to know whether or not the central nervous system could minimize energy expenditure. If there is a large possible range of energies expended for a same endpoint stiffness ellipse, then it may only be possible for experimental means such as EMG to reach strong conclusions about energy minimization. But if the range is low, then EMG measurements may not have the resolution to reveal much additional information about energy expenditure (above the information obtained by only measuring the stiffness ellipse).

## Results

### Realizable endpoint stiffness ellipses

**Fig 7:**
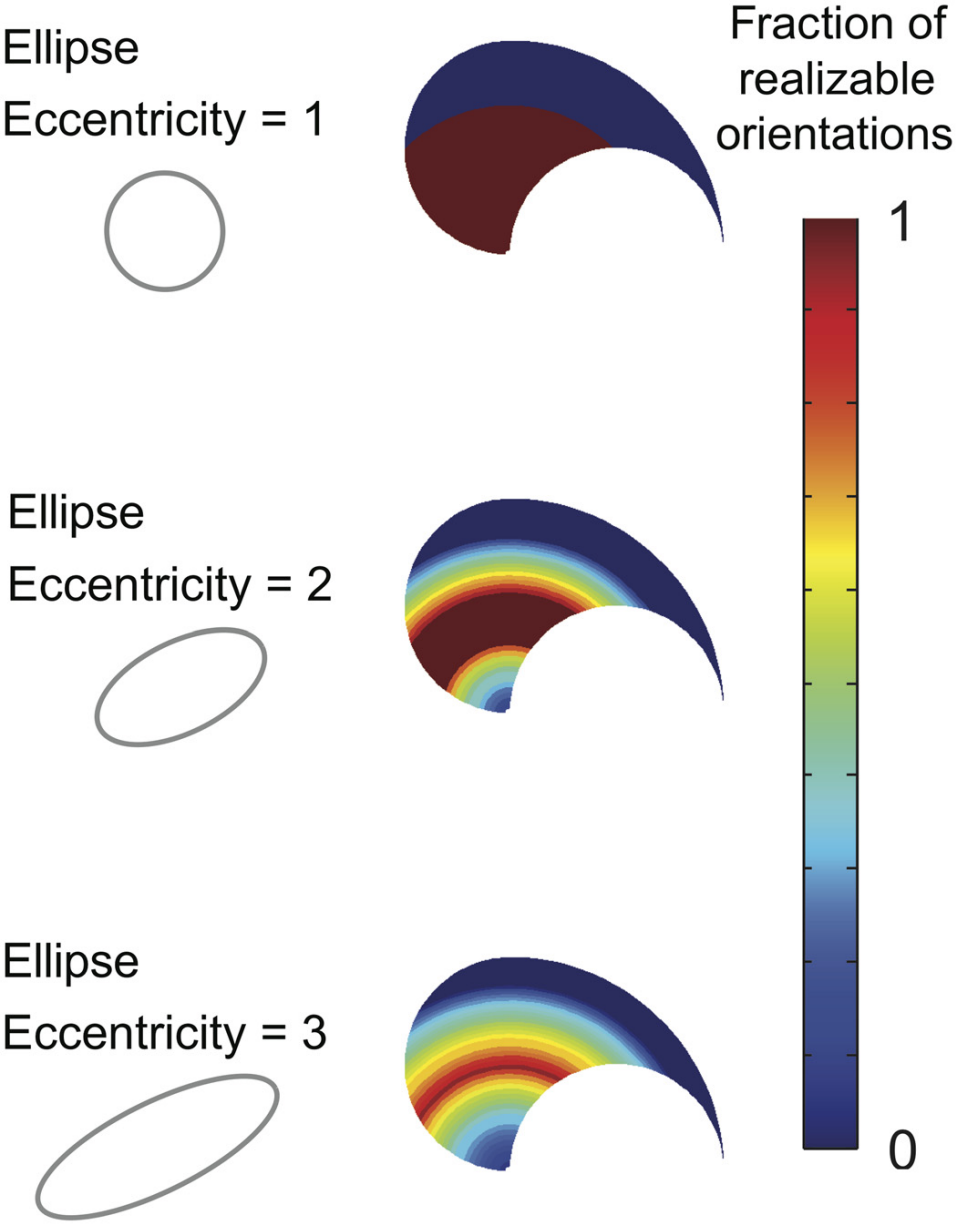
Results without synergies. The fraction of realizable stiffness orientations is heavily influenced by arm posture and stiffness ellipse shape (i.e., eccentricity measured by condition number, or ratio of length of the major to the minor axis).

Fig 7 shows the fraction of realizable endpoint stiffness ellipse orientations for various ellipse shapes throughout the workspace. We can make a couple of observations from Fig 7. First, posture has a very large effect on the range of realizable orientations (also observed in [22]). Second, the range of realizable orientations decreases with increasing ellipse eccentricity. Thus a more uniform ellipse that is closer to a circle is easier to achieve throughout the workspace, but also arguably less able to set specific directions of higher or lower stiffness. Also, our computational results for ellipse eccentricity = 1 is identical to the theoretical result determined by [8].

### Realizable orientations in the presence of synergies

**Fig 8:**
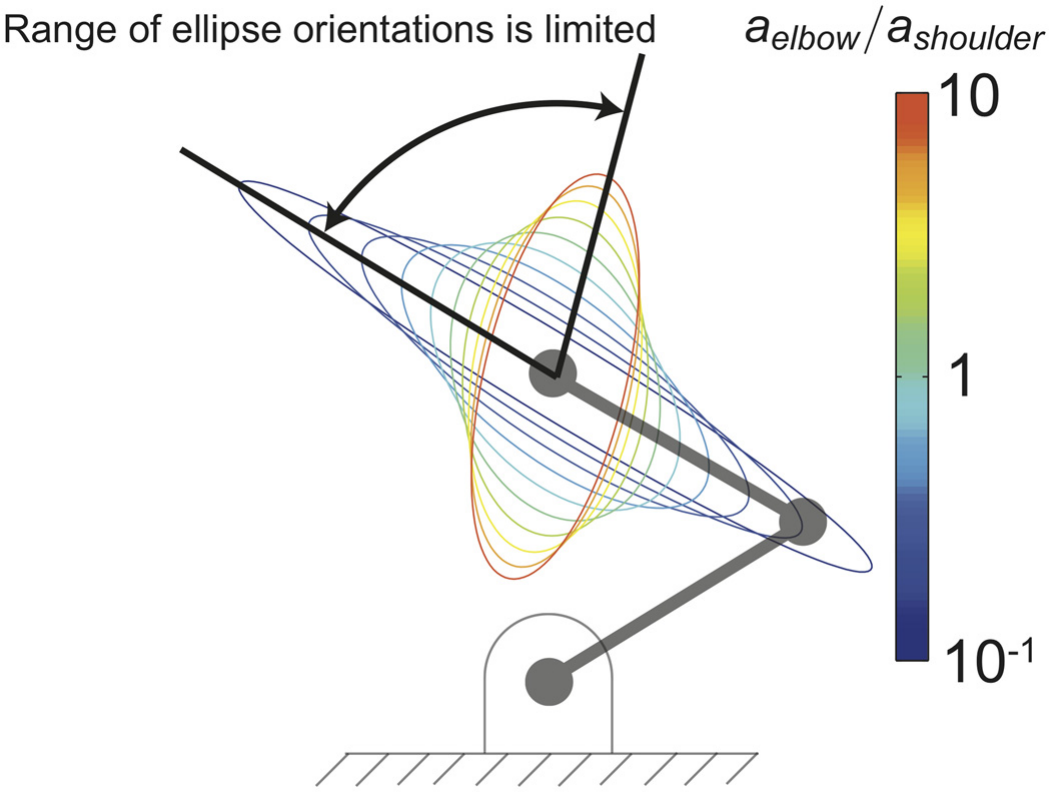
Synergies reduce the ability to control endpoint stiffness. Shoulder and elbow synergies (Fig 4) cause covariation of stiffness ellipse eccentricity with orientation and limit the range of ellipse orientations. Note that as the elbow to shoulder activation ratio changes from 10^−1^ to 10 the shape and orientation (direction of major axis) of the ellipse follow an obligatory relationship. This is expected from the fact that adding a synergy—correlated activation of multiple muscles—reduces the set of feasible activations, Fig 5.

To explore the effect of synergies in detail, we performed a more detailed analysis for a single posture. In that sample posture, Fig 8 shows the range of sizes and orientations of the stiffness ellipse achievable when varying the ratio of elbow to shoulder synergy activations from 10^−1^ to 10. The arm endpoint is in a sample *x* − *y* position (0,1), where each link of the arm has length of 1. The area of the ellipses in Fig 8 are normalized to be equal to each other to highlight the covarying shape and orientation of the stiffness ellipses.

The range of orientations is approximately 70°, which represents a realizable fraction of orientations of about 0.39. In this posture, shown in Fig 8, for all 3 ellipse shapes, the fraction of realizable orientations is 1 (all orientations are achievable) in the absence of synergies (Fig 7). In addition, we see that as the orientation of the ellipse in Fig 8 changes, the shape of the ellipse must also change. The range of physically-realizable ratios of elbow to shoulder synergy activation are likely much less extreme than two orders of magnitude, which would result in an even smaller range of possible ellipse orientations. Therefore, we see that using the synergies observed by Gomi and Osu [19] severely limits the ability of the CNS to control the shape and orientation of the endpoint stiffness of the arm.

### Energy expenditure ranges

**Fig 9:**
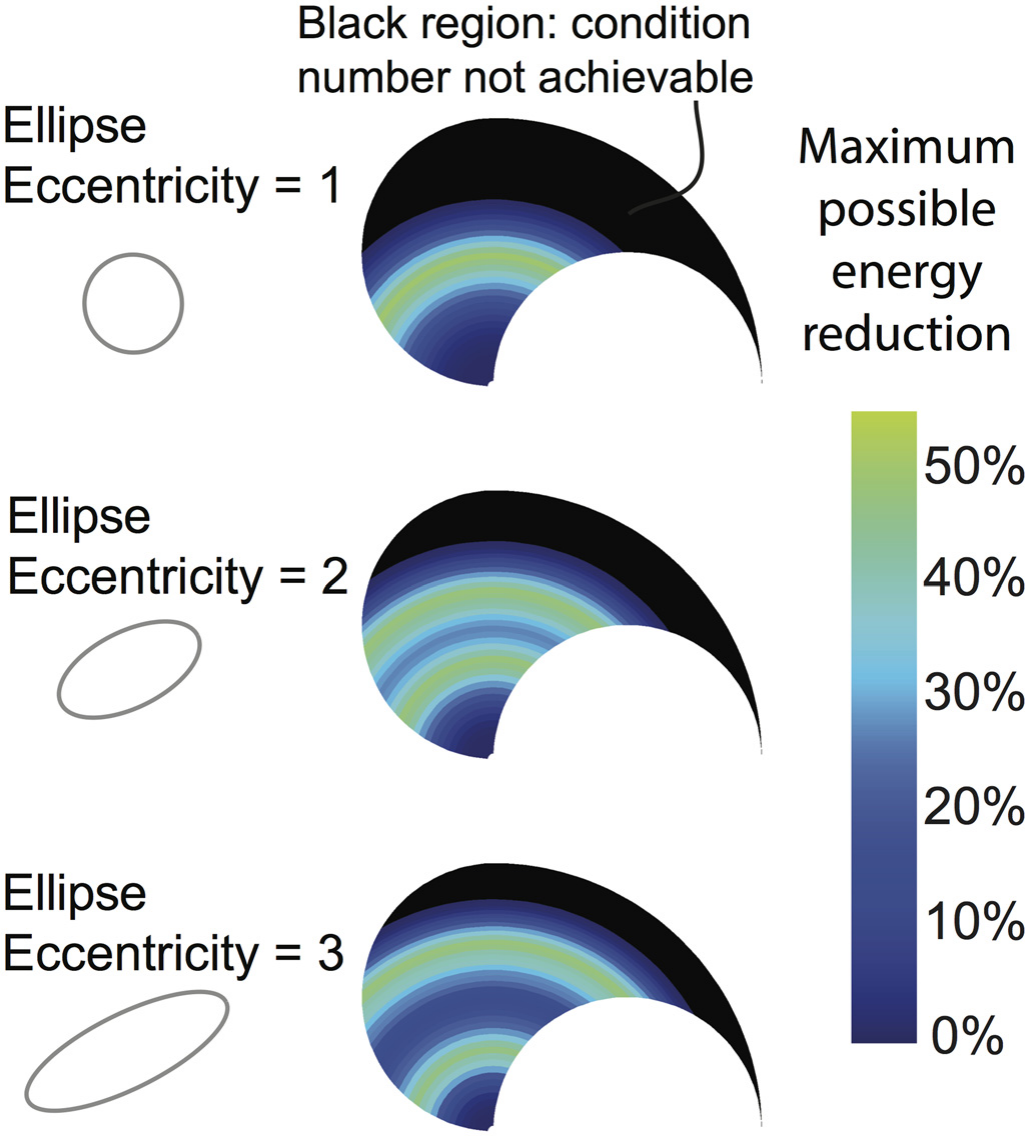
Some arm postures compromise the ability to minimize energy. Black regions indicate locations in the workspace (i.e, limb postures) where meeting the desired endpoint stiffness leaves no room to minimize energy. These regions are also heavily influenced by the desired stiffness ellipse shape (i.e., eccentricity). Even when minimizing energy is possible, that ability seldom reaches a reduction of 50% and is typically < 10–30%.

Fig 9 shows the greatest possible reduction in energy expenditure given a stiffness ellipse shape and arm posture for *any orientation*. Note the strong dependence on the posture of the arm (i.e., location in the workspace). In general, the maximal possible energy reduction for many of the workspace postures for these stiffness ellipse shapes is low (10–30%), but can increase significantly to around 50% for some specific postures.

**Fig 10:**
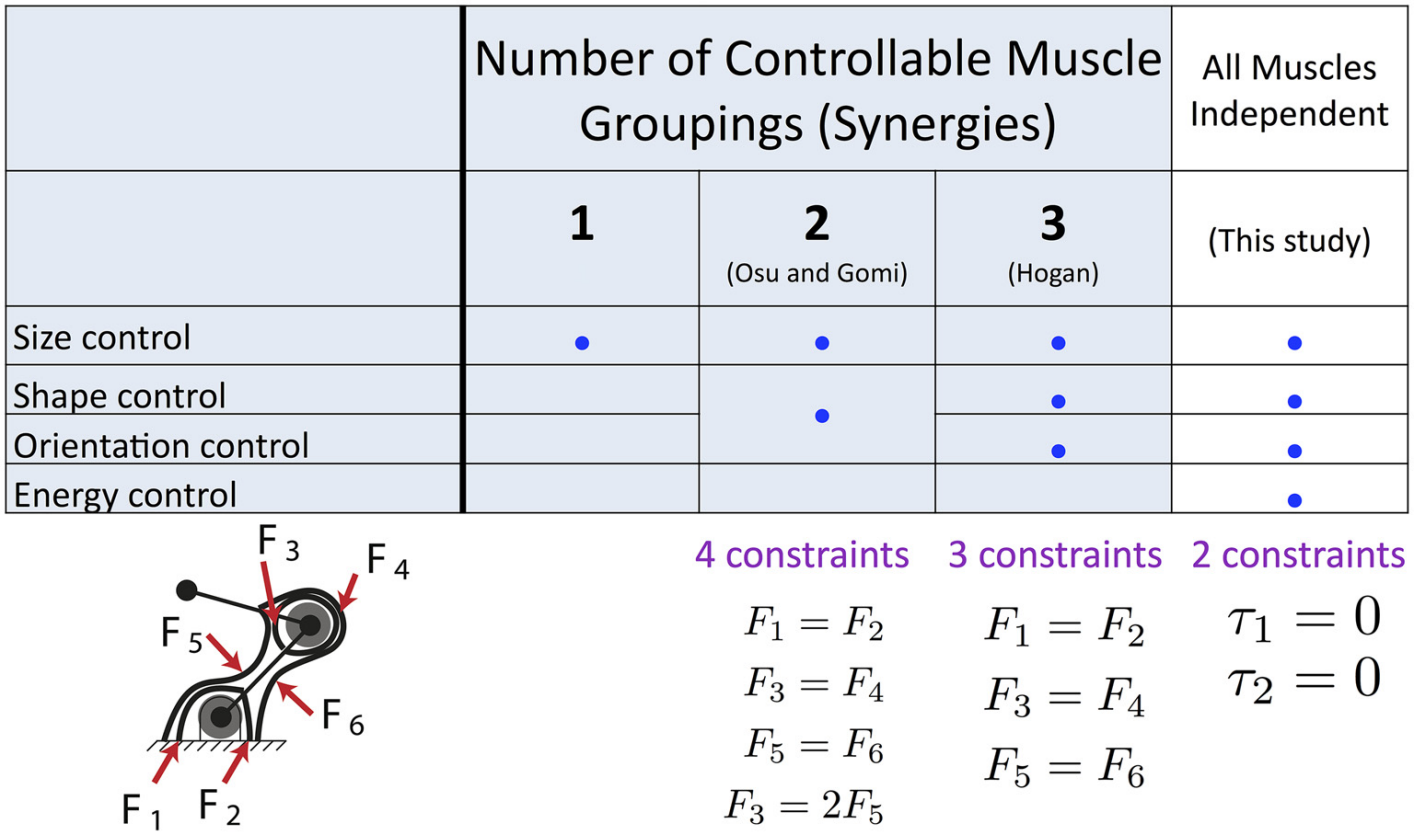
Summary of results and comparison to prior work. Implementing fewer synergies (i.e., independently controllable groupings of muscle activations) improves the independent control over the size, shape and orientation of the stiffness ellipses, as well as the energy consumption associated with each solution.

Fig 10 summarizes our findings, and compares them to prior work. We see that implementing fewer synergies (i.e., fewer muscle groupings, that reflect greater correlation among muscle activations) reduces the independent controllability of the size, shape and orientation of the stiffness ellipses, as well as the energy consumption.

## Discussion

The literature on muscle synergies is large and growing. There are already several papers debating their origins, advantages, and disadvantages [24, 28, 29, 42, 46]. The goal of this study, however, is to speak to the need pointed out by several authors to investigate the relationship between muscle synergies and the neuromechanical constraints that define a task (sometimes also called task variables) [24, 28, 34, 40, 42, 46]. We do so in the context of the neuromechanical consequences of using synergies while meeting the multiple and compounding constraints that define tasks in the ‘real world’ [34, 41, 45, 47], such as the well accepted need to regulate the stiffness of the endpoint of the arm (e.g., [1–22]).

**Fig 11:**
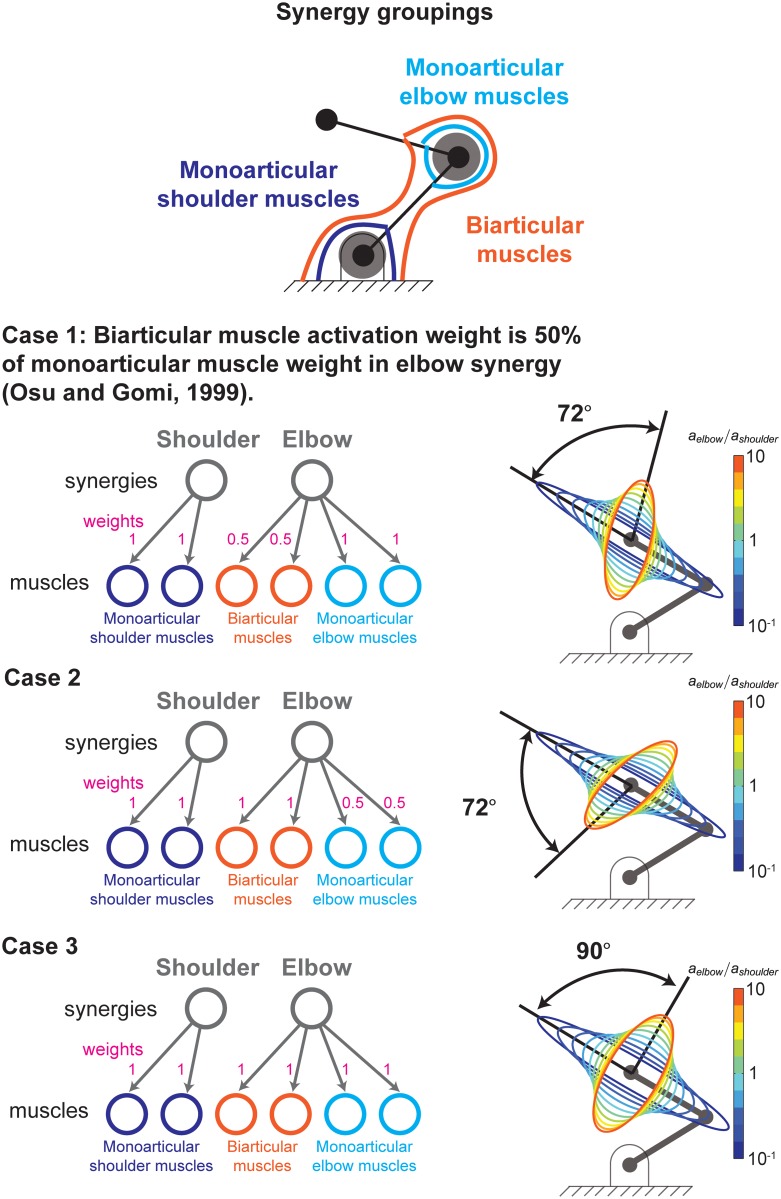
Regardless of their details, synergies bring about functional limitations, Part I. In addition to the synergies reported by Osu and Gomi (1999), we explored the functional consequences of five additional potential synergies (i.e., weights in the correlations among muscle activations). The first two are shown here as Cases 2 and 3. The remaining three are shown in Fig 12.

**Fig 12:**
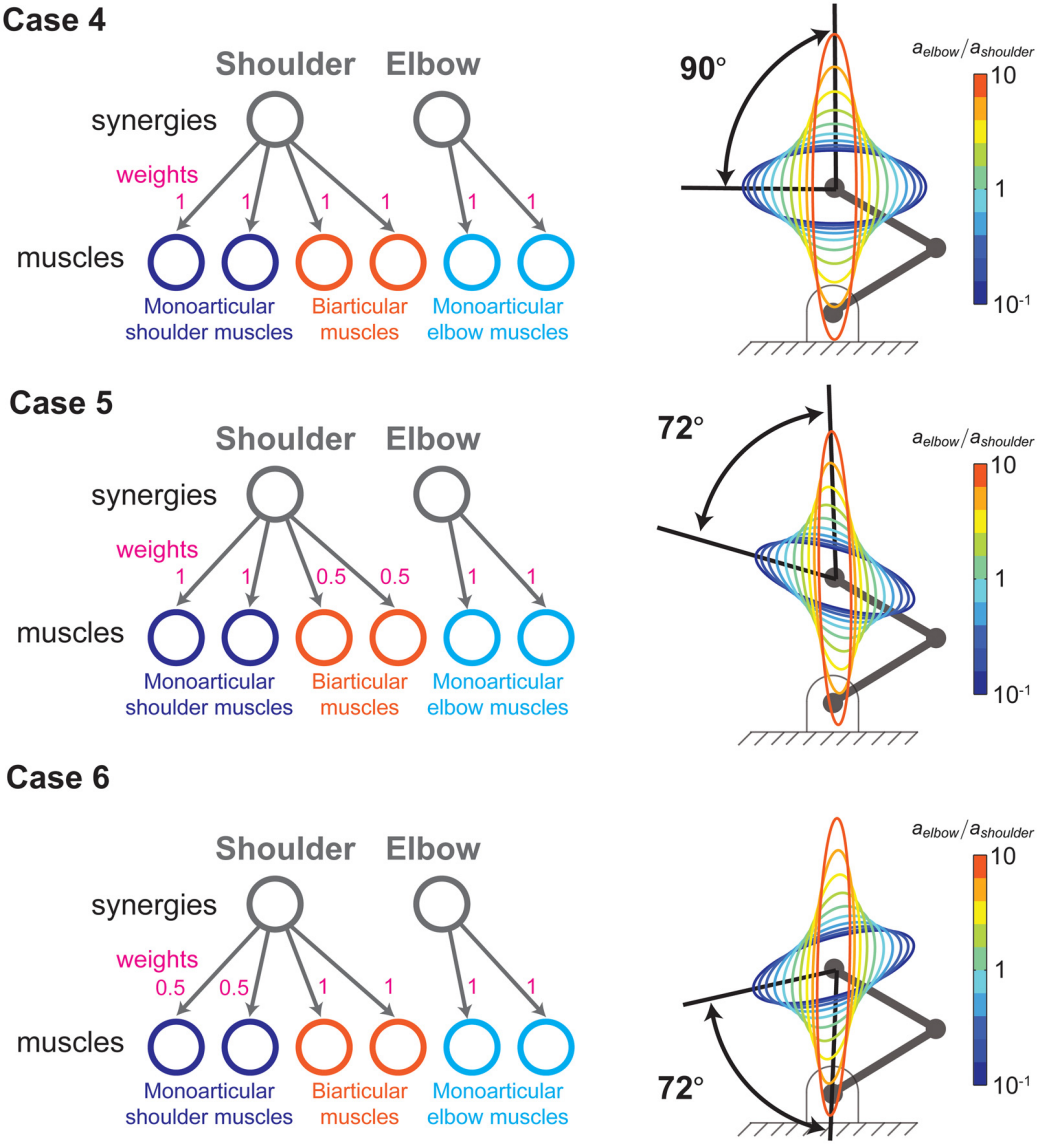
Regardless of their details, synergies bring about functional limitations, Part II. Functional consequences of three additional potential synergies, for total of five beyond those reported by Osu and Gomi (1999), as mentioned in Fig 11. Because synergies invariably imply a loss of control degrees of freedom (i.e., fewer independently controllable muscles as seen in Fig 5), it is to be expected that they all reduce the controllability of the size, shape and orientation of the stiffness ellipses as shown here and in Fig 11. Therefore, our results are generalizable to the concept of synergies in general, and are not limited to the particulars of any one specific synergy.

In the literature mentioned above, the origins of synergies as well as their specific structure and permanence continue to be debated. In their paper on static arm postures, Osu and Gomi (1999) mention that other arm synergies have been reported and that the regulation of muscle activation in static conditions seems to be quite different from that during movements. Nevertheless, this does not affect our main finding that synergies—regardless of their origin, structure or permanence—have important neuromechanical consequences in a variety of functional domains. This is because synergies imply a loss of control degrees of freedom (i.e., fewer independently controllable muscles). Therefore, the specifics of the synergies we chose to simulate as reported by Osu and Gomi do not affect the generality of our results. In fact, we went on to simulate five additional synergies as shown in Figs 11 and 12, labeled Cases 2 through 6. In all cases, synergies yield a reduction in the controllability of the size, shape and orientation of the stiffness ellipses.

It is important to mention that comparing muscle coordination and stiffness regulation in static versus dynamic movement conditions may not be advisable—or even possible. This stems from the fact that the physics and neuromuscular physiology of the control of static force versus movement are inherently distinct and can even be incompatible; see [36, 45, 48–50] and references therein. In addition to the differences in their governing equations and the force-length properties of muscle, the control of movement at a neurological level additionally requires the careful and time-sensitive orchestration of alpha-gamma co-activation and reciprocal inhibition of eccentrically contracted muscles to prevent the disruption of the movement [51]. This stems from the fact that the control of tendon excursions is overdetermined (few joint angles determine the necessary lengths of all musculotendons). This is the opposite of the underdetermined control of joint torques (many combinations of muscle forces can equivalently produce a given net joint torque) [36, 41]. Therefore, orchestrating alpha-gamma co-activation and reciprocal inhibition to produce movement imposes additional time-varying constraints that distort and reduce the feasible activation set for a given endpoint stiffness ellipse compared to the static condition. From this perspective, our results for static endpoint stiffness are a best-case scenario as the additional constraints to produce movement will likely exacerbate the limitations imposed by synergies. Understanding muscle coordination and stiffness regulation in static versus dynamic movement conditions remains an important area in motor control in need of attention [36, 48].

The feasible activation set—i.e., all feasible neural commands to achieve a given task [34, 35, 41, 43, 44]—has a well defined structure given by the biomechanics of the limb and the constraints defining the task. Muscle synergies reduce the number of independent degrees of freedom for control from the (usually large) number of independently controlled muscles, to a smaller number of independently controlled groupings of muscle activations. The presence of synergies, by reducing the number of independent degrees of freedom for control, naturally reduces the size and affects the shape of the feasible activation set—and therefore the set of tasks that are possible [34, 40].

This geometric approach uses a 6-muscle arm model with experimentally derived synergies to show that synergies severely constrain the ability to control the properties of the stiffness of the arm’s endpoint. Furthermore, it also shows reduction in the flexibility of energy consumption to implement them. That is, by reducing the dimensionality of the feasible activation set, synergies drastically limit the ability to orient the endpoint stiffness ellipse independently of its shape. The range of achievable orientations in the absence of synergies is already very sensitive to posture, but still allows significant energy minimization in some postures. Implementing synergies drastically reduces, and can even remove, the ability to minimize energy.

We would like to point out an important difference in our formulation compared with other modeling studies for arm stiffness [3, 5, 7, 8, 11, 15]. The general form of the joint stiffness matrix in these studies is (for all equal moment arms):

$$K_{joint}=\left[\begin{array}{cc} K_{s}+K_{b} & K_{b}\\ K_{b} & K_{e}+K_{b} \end{array}\right]$$ (15)

where *K*_*s*_ is the shoulder stiffness provided by co-contraction of the mono-articular shoulder muscles, *K*_*b*_ is the bi-articular joint stiffness provided by co-contraction of bi-articular muscles, and *K*_*e*_ is the elbow stiffness provided by co-contraction of mono-articular elbow muscles. This implies 3 constraints, and therefore 3 degrees of freedom for the system. However, our formulation without synergies has 4 degrees of freedom since only 2 constraints must be satisfied ($RF_{0}\vec{a}=0$).

This study analyzes the extent to which the active stiffness (i.e., not including passive or reflexive muscle stiffness) of the endpoint of a simulated arm can be controlled in the presence or absence of muscle synergies. That is, the extent to which the endpoint of the limb would displace passively in response to a force perturbation in every direction. That stiffness is the product of the level of activation of each muscle, and the anatomy and posture of the limb. We assumed, as others have in the past, that the active stiffness of a muscle is linear and proportional to the maximal force a muscle can produce and the level to which it is activated. While the linearity of active muscle stiffness with respect to muscle strength and activation is likely not entirely realistic, we focused on the effects of the presence or absence of muscle synergies. Using a nonlinear relationship would likely produce different numerical results for the precise shape, size and orientation of stiffness ellipses. However, it would not overcome the limitations that muscle synergies impose because those limitations come about from a reduction of the number of individually controllable muscles. That is a matter of scale rather than quality. Future work should naturally explore whether or not more realistic physiological mechanisms for muscle stiffness exacerbate the effects of synergies—particularly in neurological conditions. In addition, this model is limited in that it does not take into account passive muscle stiffness, reflexive stiffness, or feedback pathways, which can clearly be used to minimize energy further depending on the frequency content of a perturbation or motor noise during a task. It has been suggested [9] that some studies involving endpoint stiffness analysis may incorporate active reflex contributions [1, 5, 21]. If only active, neurally-driven, stiffness properties are considered and there is no net force at the endpoint, then the endpoint stiffness matrix is symmetric. It has been noted that any non-symmetric component of endpoint stiffness “can only be due to heteronymous inter-muscular feedback” [8]. Although future work is needed to explore these effects, our study is still able to help shed light on conflicting findings even if we only considered active stiffness without producing any net endpoint force or torque.

A subtle but important issue is that studying symmetric endpoint stiffness does not take away from our findings, bur rather enhances our result about the functional limitations of synergies. Adding a net endpoint force (or torque) deforms the symmetry of endpoint stiffness, but it also further constrains the range of stiffness modulation. Balasubramanian and colleagues have made this point well by indicating that defining an endpoint force imposes an additional set of functional constraints that compromise the modulation of endpoint stiffness [34]. Similarly, our formulation presents a best-case scenario from the perspective that we do not consider the effects of signal-dependent noise. Selen and colleagues [52] studied the general case of endpoint stiffness modulation while producing a net endpoint force vector plus a non-zero endpoint torque under different stability conditions. They demonstrate the additional control trade-offs that arise when considering the potentially destabilizing effect of signal-dependent noise.

Our model, like many others used to study arm stiffness [3, 8, 12, 13, 37], assumes equal moment arms, equal maximal muscle forces, and a planar 6-muscle arm anatomy. While this work could be easily extended to 3-D modeling and utilizing physiological values for moment arms and maximal muscle forces (as in [22]), our model includes both mono-articular and bi-articular muscles. These suffice to capture the gross capabilities of human arms since there are no bi-articular muscles that cross over from one side of the shoulder to the other side of the elbow. More importantly, the results and conclusions formed here about the effects of synergies, stiffness synthesis, and energy minimization remain the same.

Our results suggest ways in which future high-dimensional models and arm stiffness experiments may be conducted to analyze stiffness synthesis strategies used by the CNS such as synergies, energy minimization, posture adjustment, and active reflex pathways. Reaching experiments could test stiffness ellipses in various postures during the reaching movement, since stiffness ellipsoid orientation flexibility is very sensitive to small changes in posture. Findings that conflict with the results of such a study could be analyzed in more detail as this would suggest significant feedback pathways that were developed as a result of motor learning and neural plasticity. More research into muscle synergies will help elucidate existing mysteries of neuromuscular control and empower improved mechanisms for therapeutic interventions for neuromuscular disorders in aging and disease.

More generally, to our knowledge, this is the first study of the neuromechanical and energetic consequences of using synergies while meeting the multiple and compounding force and stiffness constraints that define tasks in the real world, particularly important for unstructured environments. As pointed out previously (e.g., [34, 45, 47]), we expected to see a natural reduction of task capabilities with the implementation of synergies. But understanding the specific task-level neuromechanical trade-offs in detail is critical to move our field forward. For example, Selen and colleagues [52] highlight that (even in the absence of synergies) observed stiffness geometries and their pattern of change with instability are the result of a tradeoff between maximizing the mechanical stability and minimizing the destabilizing effects of signal-dependent noise. In addition, as pointed out in [42], understanding neuromechanical trade-offs require that we distinguish between synergies that are extracted descriptively from data vs. synergies that are implemented prescriptively by a controller. The work presented here is very much taking the latter approach. We asked what feasible activation sets result when meeting a given set of task constraints with and without synergies. We find that when synergies are implemented prescriptively, the trade-off is a drastically reduced, or lost, ability to control the details of the endpoint stiffness of the arm, and the energy used to produce it.

An objection to the strength (though not to the substance) of this conclusion is that having many more muscles will naturally allow the implementation of synergies without such a drastic reduction of the feasible activation set. We agree with this interpretation as we have argued that thinking of vertebrates as having ‘too many’ muscles is paradoxical with evolutionary biology and clinical reality. That is, we have barely enough muscles for versatile and robust function in the real world [41, 45, 47]. Prescriptive synergies can and should be generalizable, flexible and learnable as correctly argued by several authors [26, 29, 46, 53], which is enabled by our many muscles.

More generally, every modeling study must assess its generalizability to everyday life—especially models with a relatively small number of joints and muscles. In such simplified systems, a few constraints may suffice to artificially deplete the system of its control degrees of freedom. The question then becomes whether, in models with many more muscles, the achievable stiffnesses are ‘good enough’ for the usual, day-to-day operation of the limb; and if so, from the functional perspective this reduced flexibility may not really be a disadvantage at all. We and others have debated this important issue in the contexts of muscle redundancy for the production of static forces, unimpeded limb motion, and their combinations [41, 45, 48, 54, 55]. Importantly, real world tasks are the subject of neuroethology, which includes the evolutionary and comparative study of the mechanistic control of natural behavior by the nervous system [56–58]. In this context, having more (or even many more) muscles than joints would be an appropriate anatomical adaptation to satisfy multiple constraints. This is because natural behavior is defined by multiple and often competing constraints, which would naturally reduce the feasible activation set (and therefore the feasible output sets) much more than the reductionist experimental tasks we often study [35, 47]. Therefore, the extent and quality of redundancy cannot be expressed simply as the number of muscles. It is the structure and dimensionality of the feasible activation set (after all relevant constrains are taken into consideration) that helps us see muscle redundancy from a neuroethological perspective. For a critical review of the classical concept of muscle redundancy see [36]. For example, in the case of producing active endpoint stiffness during limb movement while manipulating an object, the nervous system must issue neural commands, coordinated throughout the entire duration of the movement, to at the very least simultaneously:

- Set the necessary endpoint stiffness size, shape and orientation [19]
- Specify the direction, speed and duration of the movement [1]
- Control for the desired endpoint forces and torques [34]
- Consider the influence of motor noise [52]
- Regulate activity across the *α*-motoneuron pools to produce the necessary joint torques as per the classical muscle redundancy force-sharing problem (e.g., [39, 43])
- Coordinate reciprocal inhibition of *α*-motoneuron pools across shortening and lengthening muscles [59]
- Tune *γ*-drive and/or inhibit the stretch reflex in muscles undergoing eccentric contractions (e.g., [51, 60])
- Mediate interneuronal interactions [61]
- Satisfy the temporal constraints of conduction velocities, muscle excitation-contraction dynamics, and activation/deactivation time constants [62] to ensure the continuity of these neural commands as the motion progresses

This compounding of multiple, potential conflicting, spatial and temporal constraints naturally leads to a dramatic shrinking of the set of feasible neural commands for natural movements even when we have many muscles. This line of thinking helps clarify the apparent and longstanding paradox between the classical concept of muscle redundancy and the clinical reality of motor development and dysfunction [36]. For example, clinicians have long been aware of how disorders of reflexes or the neural circuits of ‘afferented muscles’ lead to disruptions or failures of everyday movements and interactions with objects (for an overview see [63, 64]). Thus, these pathologies of everyday limb function may in fact be a natural consequence of the nervous system failing to meet the multiple and stringent spatio-temporal demands listed above—in spite of having many muscles.

In spite of the simplified model used in the prior literature and here, our results nevertheless add to current thinking about endpoint stiffness in two critical ways. First, they enable us to explore the specific task-level trade-offs associated with specific synergies. And second, they show that there is a natural limit to how generalizable and flexible any synergy can be. Simply said, every synergy that is prescribed will reduce the feasible activation set (and thus the set of feasible actions) as strictly as the mechanics of the limb or the constraints of the task.

Thus if one is to prescribe synergies to meet the multiple constraints of the many tasks we face in real life, how many synergies should one learn? The idea that each prescribed synergy solves, by construction, a well-defined control problem is well studied in the control literature [42, 65, 66]. We propose that the approach presented here will enable future research to understand the extent to which organisms find the middle ground between prescribing synergies to simplify control (at the expense of loss of functionality) and retaining the independence of muscle control to enable the learning, execution and refinement of motor function that meets the multiple and competing demands of tasks in the real world.

## Supporting Information

S1 Dataset Simulation data. (XLSX)
